# Evaluation of Eruption of Permanent Teeth in Beagle Dog Extraction Sites Filled with Carbonate Apatite

**DOI:** 10.3390/ma16247624

**Published:** 2023-12-13

**Authors:** Toshiro Kibe, Kenta Nakazono, Kaoru Yamashita, Ryohei Tada, Yusuke Ono, Kiyohide Ishihata

**Affiliations:** 1 Department of Oral and Maxillofacial Surgery, Field of Oral Maxillofacial Rehabilitation, Developmental Therapeutics Course, Graduate School of Medical and Dental Sciences, Kagoshima University, 8-35-1, Sakuragaoka, Kagoshima 890-8544, Japan; zono@d1.dent.kagoshima-u.ac.jp (K.N.); r-tada08@d1.dent.kagoshima-u.ac.jp (R.T.); y1992325@d1.dent.kagoshima-u.ac.jp (Y.O.); ishihata@dent.kagoshima-u.ac.jp (K.I.); 2 Department of Dental Anesthesiology, Field of Oral Maxillofacial Rehabilitation, Developmental Therapeutics Course, Graduate School of Medical and Dental Sciences, Kagoshima University, 8-35-1, Sakuragaoka, Kagoshima 890-8544, Japan; kaorun@dent.kagoshima-u.ac.jp

**Keywords:** alveolar cleft, bone grafting material, carbonate apatite, permanent teeth eruption

## Abstract

Autologous bone grafting is the primary method for treating alveolar clefts. However, bone grafting materials are desired as alternatives to autogenous bone to reduce surgical invasiveness. Here, we present an animal study evaluating the effect of carbonate apatite (CA) on the spontaneous eruption of permanent teeth. The bone grafting materials included CA, natural bovine bone (BB), and hydroxyapatite (HA). In 15 8-week-old male beagle dogs, the left mandibular deciduous premolars (DP) two and three were extracted and subsequently filled with CA, BB, and HA. The animals were euthanized after a predetermined number of days, and samples were collected for microcomputed tomography and histological evaluation. Spontaneous eruption of the succeeding permanent teeth (P3 and P4) was observed in the CA group at 14 weeks. Delayed eruption of the succeeding permanent teeth was observed in the BB and HA groups. CA could serve as a viable alternative to autogenous bone for treating alveolar clefts.

## 1. Introduction

Cleft lip and palate (CLP) is a common maxillofacial congenital malformation often associated with alveolar clefts. CLP may be caused by a combination of factors, including genes, external factors that the mother comes into contact with in her environment, mother’s diet, and certain medications that the mother uses during pregnancy [1,2]. However, the detailed pathogenesis of CLP remains unclear. The frequency of CLP varies by race, but it is estimated that approximately 1 in 600 to 1000 children are born with CLP. Therefore, the optimization of CLP treatment would be of great significance. Although there are many different types of CLP, many patients with CLP also have alveolar clefts. Alveolar clefts in CLP patients adversely affect permanent tooth eruption and maxillofacial growth [3]. Reconstruction of the alveolar cleft is important in the treatment of CLP [4,5]. The purpose of autogenous bone grafting is to fill large alveolar bone defects and achieve physiological function similar to that of normal bone. In general, autogenous bone grafting into the alveolar cleft promotes the eruption of permanent teeth and stabilizes the maxilla [6,7]. Autologous bone grafting is generally the first choice for alveolar cleft reconstruction [3,6,8,9]. However, owing to the invasive nature of the bone harvesting site and the limited amount of obtainable bone, the demand for bone replacement materials as an autogenous bone alternative is on the rise. In addition, this surgery may reduce the patient’s quality of life due to postoperative exercise restrictions and prolonged hospitalization [10]. Crucial aspects of alveolar cleft reconstruction include creating substantial bone connections at the defect site and ensuring unobstructed permanent tooth eruption. Therefore, the chosen bone graft material for alveolar cleft reconstruction should possess strong bioabsorbable properties to prevent interference with the natural eruption of permanent teeth [11].

Bone is a composite composed of carbonate apatite [CO3Ap; Ca10-a(PO4)6-b(CO3)c], which is an inorganic component, and organic components, including collagen [12]. Carbonate apatite (CA) is an inorganic component of bone, and CA-based bone grafting materials are bioabsorbable and are considered a part of the bone glue modeling cycle in vivo [13]. Moreover, previous reports have shown that low-crystalline CA exhibits better osteoinductive properties than hydroxyapatite (HA) [13,14]. Furthermore, another study reported that carbonate content increases osteoclastogenesis and substrate resorption [15]. In dental practice, CA is often used as a bone substitute material for implant surgery; it implant placement with sinus floor augmentation using CA reportedly results in almost complete resorption of CA and its replacement by new bone in approximately 8 months [16]. In addition, the presence of both mature and osteoid bone has been observed, and active bone remodeling has been reported [17]. Many studies of CA in dental practice have been reported in relation to implant surgery [18,19,20]. There are several resorbable bone grafting materials, but nothing has been actively used in the treatment of alveolar clefts [7]. Recently, there have been reports of bioabsorbable hydroxyapatite being applied to secondary bone grafting of unilateral alveolar clefts (Sakamoto et al., 2020. [21]). Recently, we reported the use of octacalcium phosphate (OCP) for secondary bone grafting in unilateral alveolar clefts [11,22]. In dentistry, bioactive glass has also been reported as a useful biomaterial [23]. However, to the best of our knowledge, no reports exist on CA bone grafting materials that have documented the spontaneous eruption of permanent teeth.

This study aimed to utilize CA bone replacement material to fill the extraction socket of a deciduous tooth in a male beagle dog by observing the spontaneous eruption of the succeeding permanent tooth. Additionally, this study aimed to histologically observe osteogenesis and the residual state of the bone materials.

## 2. Materials and Methods

### 2.1. Ethics

This study was approved by the Division of Laboratory Animal Science at the Natural Science Center for Research and Education at Kagoshima University (No. D19041) and performed according to the Japanese Government Animal Protection and Management Law.

### 2.2. Animals

We used 8-week-old male Toyo beagle dogs (Kitayama Labes Co., Ltd., Nagano, Japan) as a wound healing model in this study. The dogs were acclimated to our experimental environment for one week before experimentation and housed in individual cages. The dogs were maintained and treated according to the protocols of the Division of Laboratory Animal Science at the Natural Science Center for Research and Education at Kagoshima University.

### 2.3. Bone Grafting Materials

Three commercially available bone substitutes were used in this study. The materials included CA (Cytrans^®^ Granules, GC Corporation, Tokyo, Japan) [24,25], natural bovine bone (BB) (Geistlich Bio-Oss^®^, Geistlich Pharma Japan, Tokyo, Japan), and HA (NEOBONE^®^, Aimedic MMT Co., Ltd., Tokyo, Japan). These bone grafting materials were granular in form. BB and HA served as comparative controls.

### 2.4. Animal Experiments

**Figure 1:**
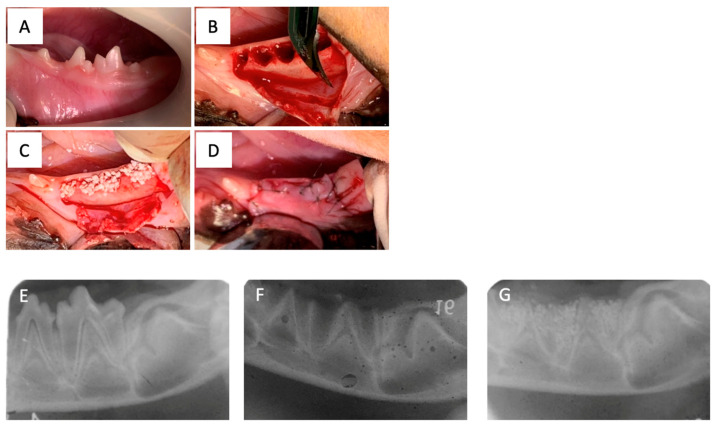
Treatment was performed as shown in (**A**–**D**) and confirmed by radiographs in (**E**,**F**). (**A**) Untreated left lateral mandibular molars (DP3 and DP4). (**B**) Post-extraction of the left mandibular molars (DP3 and DP4). (**C**) Bone graft material at the extraction site. (**D**) Closure of the grafted area of the bone grafting material. (**E**) Dental X-rays before treatment. (**F**) Dental X-rays at the extraction site. (**G**) Dental X-rays after treatment.

All animal experiments were performed at the Animal Technology Laboratory of the Tsukuba Research Center, Hamri, Inc., Ibaraki, Japan. Anesthesia and X-rays were performed. Beagle dogs were anesthetized intramuscularly with a mixture of ketamine hydrochloride and xylazine in equal volumes at a dosage of 0.4 mL/kg. The same solution was used for anesthesia maintenance. Monitoring of oxygen saturation, electrocardiography, and the autonomic nervous system were performed throughout the anesthesia. Under the above anesthesia, images were taken with a digital X-ray DX-I at 55 KV, 0.16 mAs from one direction. Dental X-ray films were used for image development and fixation using bath solutions. The implantation site for the bone grafting material was secured. The area around the second and third premolars in the left mandible was disinfected with veterinary isodine, and local anesthesia was administered using 0.3 mL of dental xylocaine (lidocaine hydrochloride 6 mg and adrenaline 0.00375 mg). Surgical procedures and the filling of A were performed in accordance with previous reports [26]. In brief, the gingiva in the same area was incised and debrided, and the premolars (DP2 and DP3) were extracted. The placement of the bone grafting materials involved the utilization of three types of materials: CA, BB, and HA. Fifteen beagle dogs were used in this study. These materials were applied in distinct groups: CA group (CA on the left side, untreated on the right side), BB group (BB on the left side, untreated on the right side), and HA group (HA on the left side, untreated on the right side) (Table 1). After implantation, dental X-rays were obtained using the method described above. After a reduction incision was made in the gingiva, the wound was sutured using 6–0 nylon (Figure 1). Subsequently, dental X-rays and intraoral photographs were taken under anesthesia at 4 and 7 weeks and at the time of specimen collection for observation.

### 2.5. Postoperative Follow-up

Postoperative follow-up was conducted daily, and antibiotics (enrofloxacin) were administered intramuscularly at a rate of 0.4 mL/10 mg/kg for at least 3 days after surgery to prevent infection. Buprenorphine hydrochloride injection (0.2 mg/kg) was administered subcutaneously once daily for at least 3 days as an analgesic. The animals were weighed daily, and if they experienced a considerable wound infection or a noticeable decline in their drinking or eating habits resulting in a 20% loss of body weight, they were humanely euthanized through an overdose of injectable thiamylal sodium (50 mg/kg or more administered intravenously). The specimens collected included jawbone tissue from dogs euthanized in this manner.

All groups were observed using X-rays at 4, 7, and 14 weeks after treatment. The animals were euthanized after a predetermined number of days (Table 1), and samples were collected for microcomputed tomography (CT) (inspeXio SMX-100CT, Shimadzu, Kyoto, Japan) and histological evaluation. For histological analysis, hematoxylin–eosin (HE) staining was performed to observe morphology and tartrate-resistant acid phosphatase (TRAP) staining was performed to observe osteoclasts. Tissue specimens were observed under an optical microscope (BZ-X710; Keyence, Tokyo, Japan) [27].

The cadavers were stored in a freezer and incinerated at the Animal Technology Laboratory of the Tsukuba Research Center, Hamri, Inc.

### 2.6. Micro-CT Analysis

In micro-CT analysis, the following parameters were measured using the plane orthogonal to the tooth axis of the premolars and the cortical bone surface at the lower end of the mandible as the reference plane. At sites P3 and P4, (1) the height from the mandibular inferior margin to the respective alveolar crest, (2) the height from the mandibular inferior margin to the P3 or P4 cusp, and (3) the thickness of the remaining bone material were measured. The OsiriX image analysis software (v.4.1.2 macOS; https://www.osirix-viewer.com/osirix/osirix-md/ (accessed on 10 February 2021)) (OsiriX, Pixmeo SARL, Geneva, Switzerland) was used for measurements [22].

### 2.7. Statistical Analysis

The significance of measurement differences between groups was evaluated using one-way analysis of variance (ANOVA) and Tukey’s multiple comparison test. All statistical analyses were performed using GraphPad Prism version 8 for Mac (GraphPad Software, San Diego, CA, USA). Statistical significance was set at *p* < 0.05. To mitigate surgeon bias in animal experimentation, a single examiner (K.N.), uninvolved in the animal experiments, assessed the CT images.

## 3. Results

### 3.1. Comparison of the Spontaneous Eruption of Permanent Teeth with Different Bone Materials

**Figure 2:**
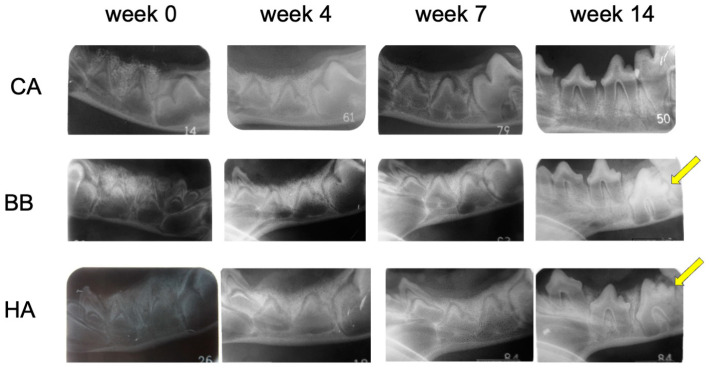
Dental X-rays at 0, 4, 7, and 14 weeks after treatment using CA, BB, and HA. Yellow arrows indicate permanent teeth with delayed eruption. CA, carbonate apatite; BB, bovine bone; HA, hydroxyapatite.

At 14 weeks, spontaneous eruptions of P3 and P4 were observed in the untreated right mandible of all groups. In the dental radiographs, granules of bone filler were observed in all groups up to 7 weeks, and no difference was observed in the degree of permanent teeth eruption. In the left mandible, spontaneous eruption of the succeeding permanent teeth (P3 and P4) was observed in all members of the CA group at 14 weeks (Figure 2). In the BB and HA groups, delayed eruption of the succeeding permanent teeth was observed. At 14 weeks, most of the granules were resorbed only in the CA group, and the CA was replaced by new bone (Table 2).

### 3.2. Evaluation of Extraction Socket Bone Formation in Different Bone Materials

**Figure 3:**
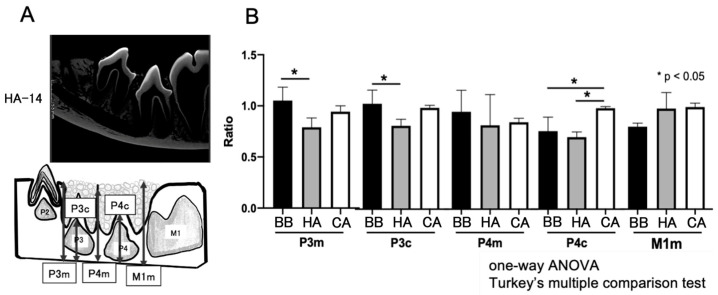
Measurement of alveolar bone height using micro-CT. (**A**) Micro-CT measurement area. (**B**) Measurements of the distance from the inferior margin of the mandible to the alveolar apex at 14 weeks after treatment, expressed as a ratio of the untreated side. CT, computed tomography; ANOVA, analysis of variance.

The extent of new bone formation was evaluated by measuring the distance between the mandibular inferior margin and the upper alveolar bone. In the micro-CT measurement results, the ratio of the untreated group was calculated as 1. Compared to the HA and BB groups, the distances of P3c and P4c in the CA group from the inferior margin of the mandible were comparable to those of the untreated side (Figure 3). However, compared with the BB and HA groups, the spontaneous eruption of P4 in the CA group was observed to be significantly more erupted.

### 3.3. Observation of the Remaining Bone Materials

**Figure 4:**
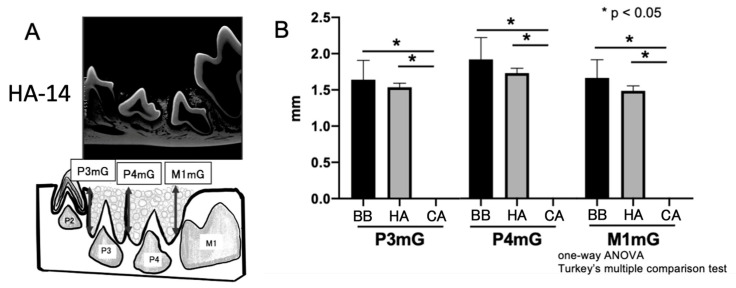
Measuring the height of remaining bone grafting materials using micro-CT. (**A**) Micro-CT measurement area. (**B**) Measuring the height of remaining bone grafting materials at 14 weeks. ANOVA, analysis of variance; HA, hydroxyapatite.

Micro-CT observations revealed the remaining bone material as granules. Compared to the HA and BB groups, no residual bone filler was observed in the CA group at 14 weeks (Figure 4). In contrast, permanent tooth eruption was impaired in areas where residual bone materials were observed.

### 3.4. Histological Observations around Bone Materials

**Figure 5:**
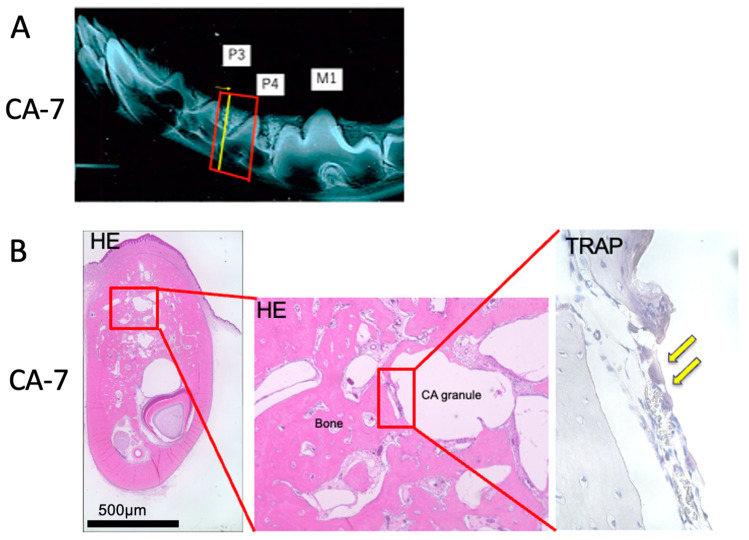
Histological observations on the seventh day after CA treatment. (**A**) Slice from which the tissue specimen was taken. (**B**) Tissue images of hematoxylin–eosin and tartrate-resistant acid phosphatase staining. Yellow arrows indicate osteoclasts. CA, carbonate apatite.

To examine the role of osteoclasts in CA resorption in vivo, samples from the CA group were histologically examined using TRAP staining (Figure 5). Many osteoclasts were observed surrounding the granules of the bone grafting material in the CA group.

## 4. Discussion

With CA, the eruption of permanent teeth in beagle dogs was observed without significant interference with their natural eruption. In our preliminary study, spontaneous sprouting of P2 and P3 was observed at approximately 22 weeks of age in the beagle dogs used in this study. Therefore, the observation period for this study using 8-week-old beagle dogs was 14 weeks. In this study, after 14 weeks, CA was completely absorbed around the eruption site of the permanent teeth and did not impede their natural eruption. When using CA in children with alveolar clefts, the natural eruption of permanent teeth must not be disturbed. In previous studies, several artificial materials have been used for alveolar bone grafting in patients with CLP. However, to our knowledge, no study has observed spontaneous permanent teeth eruption after alveolar cleft reconstruction using only artificial materials.

The efficacy of alveolar bone reconstruction in patients with alveolar clefts that was performed with alternative/complementary bone grafting materials has been reported in several previous studies. However, in these reports, permanent tooth eruption failure was frequently observed at the site of bone grafting material placement [28]. Several studies on alveolar bone reconstruction using cytokines, such as bone morpho-genetic protein (BMP)-2, which is known to be a potent osteogenic factor, and other synthetic materials have also been reported [29,30]. In these reports, BMP-2 has been reportedly promotes bone bridge formation in the alveolar cleft not alone but in combination with various bone replacement materials, such as absorbable collagen sponges.

Bioabsorbable materials such as bone graft materials are often collagen composites [31]. Because most collagen is derived from animals such as bovines and pigs, its use is expected to be difficult for religious reasons. For example, it is well known that Islam, which accounts for 23% of the world’s population (1.6 billion people), prohibits the consumption of pork and its products [32]. CA bone graft material does not contain collagen complexes and is therefore considered to be a viable bone graft material for use in many countries. Therefore, it could potentially be used worldwide in the treatment of alveolar clefts in CLP patients.

The osteogenesis of alveolar bone defects using CA bone materials has been reported in clinical trials [16,17]. Bone apatite is CA containing carbonate in its structure. Therefore, carbonate content is considered to be one of the factors governing the osteoconductivity of apatite-based bone replacement materials. It has been reported that CA bone replacement materials with relatively high carbonate content in the apatite structure have been resorbed over time [14]. Furthermore, CA, which constitutes approximately 7% of bone mass as an inorganic component, is thought to undergo bone resorption through the action of osteoclasts [33]. This study revealed osteoclasts on the surface of the remaining CA bone grafting materials at 4 weeks after treatment, and by 14 weeks after treatment, all the CA was absorbed, suggesting that CA participates in bone remodeling and serves as a scaffold for bone formation. Previous studies on rabbit femur and dog alveolar bone defects have reported that CA granules form bones faster than other bone materials [14,24]. In the spontaneous eruption of the succeeding permanent teeth (P3 and P4) after 14 weeks in the CA group, it was important for CA to be absorbed into bone remodeling by osteoclasts. Bone remodeling is the process by which old bone or autogenous bone grafts are replaced by new bone [33]. Osteoclasts play an important role in bone remodeling, as they absorb old or autogenous bone, followed by osteoblasts that form new bone. The activity of osteoclasts depends on the type of apatite. CA dissolves under weakly acidic conditions and is resorbed by osteoclasts. However, HA is barely soluble in weakly acidic conditions and is not easily resorbed by osteoclasts [34]. In this study, osteoclasts were observed on the surface of CA granules. These findings suggest that almost all CA granules were replaced by new bone before the spontaneous eruption of the succeeding permanent teeth (P3 and P4), which did not prevent the spontaneous eruption of the permanent teeth.

In addition, previous studies have reported that the CO_3_ content in apatite structures correlates well with the percentage of new bone in the overall bone defect [14]. Cytrans, the CA bone material used in this study, contains 12.0 ± 0.6 mass% CO_3_ and was reported to have the highest bone formation rate of the three apatite bone substitutes. Bio-Oss, containing 5.5 ± 0.2 mass% CO_3_, and NEOBONE, containing 0.1 ± 0.1 mass% CO_3_, were reported to have the second highest bone formation rate. In this study, bone formation rates were not directly analyzed. However, a high number of osteoclasts were observed in the carbonated apatite bone material. This result suggests that the bone formation rate of CA bone material was faster than that of other bone filling materials, leading to successful spontaneous permanent teeth eruption.

Autogenous bone grafting is commonly used to treat bone defects in alveolar clefts [3,4,5]. The most common site for autogenous bone harvesting is the iliac crest bone. Obtaining autogenous bone from the iliac crest can result in temporary mobility impairment, prolonged surgery, and reduced quality of life due to donor site pain [35,36]. Therefore, the development of artificially manufactured bone grafting materials with functions equivalent to those of autogenous bone is crucial for improving patients’ quality of life. This study suggests that CA bone substitutes hold promise as bone grafting materials for CLP treatment. In the future, long-term follow-up and the combination of implant placement and bone grafting must also be investigated [37].

This study had several limitations. Firstly, comparative observations were not performed in the same individuals with extraction-only and bone grafting material implantation procedures. If bilateral mandibular molars were extracted and treated for this study in the same individuals, the dogs would have lost weight due to reduced masticatory ability, which could have affected the experiment. Secondly, this study focused solely on evaluating hard tissue at the bone filler grafting site, with no soft tissue assessment. Because the purpose of this study was to evaluate the eruption of permanent teeth, we did not focus on soft tissue evaluation. Soft tissue evaluation is an issue for future study. The third limitation is that we did not perform a detailed comparative analysis between CA and other bone materials. The main objective of this study was to investigate the effect of CA bone materials on spontaneous permanent teeth eruption. Therefore, a detailed comparison with other bone materials was not performed. The last limitation is that the extraction sockets filled with bone materials may not have had the same volume. Although the beagle dogs used in the experiment have the same age in weeks, there could have differences in the volumes of the extraction sockets.

## 5. Conclusions

This study observed the spontaneous eruption of succeeding permanent teeth without delay at the CA bone grafting material site. These results suggest that CA bone grafting material can be a viable alternative to autogenous bone for treating alveolar clefts.

## Figures and Tables

**Table 1:** Allocation of beagle dogs to CA, BB, and HA groups for observation over specified days.

Group	Material		Period	Number
	R	L		
BB-14	Untreated	BB	14 weeks	3
HA-14	Untreated	HA	14 weeks	3
CA-4	Untreated	CA	4 weeks	3
CA-7	Untreated	CA	7 weeks	3
CA-14	Untreated	CA	14 weeks	3

**Table 2:** List of permanent tooth eruption for all groups after 14 weeks. Rated on a scale of 1, 2, or 3. 1: Unerupted or partially erupted teeth. 2: Half of the tooth has erupted compared to the untreated side. 3: The same level of eruption as on the untreated side (complete eruption).

	CA			BB			HA		
Identification Number	#7	#8	#9	#4	#5	#6	#1	#2	#3
P3	3	3	3	1	2	1	2	3	2
P4	3	3	3	1	1	1	1	1	1

## Data Availability

All data in this paper are presented in the form of figures and tables. Anyone who is interested in the specific numbers from the figures can contact the corresponding author (s2000@dent.kagoshima-u.ac.jp) directly.
